# Specific Role for GSK3α in Limiting Long-Term Potentiation in CA1 Pyramidal Neurons of Adult Mouse Hippocampus

**DOI:** 10.3389/fnmol.2022.852171

**Published:** 2022-06-17

**Authors:** Aeen Ebrahim Amini, Tsukiko Miyata, Gang Lei, Fuzi Jin, Elizabeth Rubie, Clarrisa A. Bradley, James R. Woodgett, Graham L. Collingridge, John Georgiou

**Affiliations:** ^1^Lunenfeld-Tanenbaum Research Institute, Mount Sinai Hospital, Sinai Health, Toronto, ON, Canada; ^2^Department of Physiology, University of Toronto, Toronto, ON, Canada; ^3^Department of Medical Biophysics, University of Toronto, Toronto, ON, Canada; ^4^Neurosciences and Mental Health Program, Peter Gilgan Centre for Research and Learning, The Hospital for Sick Children, Toronto, ON, Canada; ^5^TANZ Centre for Research in Neurodegenerative Diseases, University of Toronto, Toronto, ON, Canada

**Keywords:** CA3-CA1 synapses, LTP (long term potentiation), synaptic plasticity, GSK-3 (glycogen synthase kinase 3), conditional knock out mice, synaptic function, GSK-3α, GSK-3β

## Abstract

Glycogen synthase kinase-3 (GSK3) mediates phosphorylation of several hundred proteins, and its aberrant activity is associated with an array of prevalent disorders. The two paralogs, GSK3α and GSK3β, are expressed ubiquitously and fulfill common as well as unique tasks throughout the body. In the CNS, it is established that GSK3 is involved in synaptic plasticity. However, the relative roles of GSK3 paralogs in synaptic plasticity remains controversial. Here, we used hippocampal slices obtained from adult mice to determine the role of each paralog in CA3−CA1 long-term potentiation (LTP) of synaptic transmission, a form of plasticity critically required in learning and memory. Conditional *Camk2a* Cre-driven neuronal deletion of the *Gsk3a* gene, but not *Gsk3b*, resulted in enhanced LTP. There were no changes in basal synaptic function in either of the paralog-specific knockouts, including several measures of presynaptic function. Therefore, GSK3α has a specific role in serving to limit LTP in adult CA1, a postsynaptic function that is not compensated by GSK3β.

## Introduction

Glycogen synthase kinase-3 (GSK3) is a serine/threonine kinase that plays a role in a diversity of intracellular signaling pathways and biological activities in development and throughout adulthood. Dysregulation of GSK3 is implicated in many major diseases, including cancer, inflammation, diabetes, cardiac hypertrophy and an array of CNS disorders (see reviews by Beurel et al., 2015; Ahmad and Woodgett, 2020; Hajka et al., 2021). In terms of brain disorders, GSK3 has been strongly implicated in, for example, sleep disorder, psychiatric illness, neurodevelopmental disorders including autism, intellectual disability, epilepsy and neurodegenerative disorders, in particular Alzheimer’s disease (see reviews by Jaworski et al., 2019; Jaworski, 2020; Silva-García et al., 2020; Rizk et al., 2021; Sayas and Ávila, 2021). Investigating the roles of GSK3 is therefore central to the understanding of how its dysregulation may contribute to a wide array of serious conditions.

Two distinct genes encode for GSK3α and GSK3β, paralogs expressed in most tissues (Woodgett, 1990). These two isoenzymes have both overlapping and discrete intracellular localization, are constitutively active and phosphorylate numerous substrates (see review by Kaidanovich-Beilin and Woodgett, 2011; Beurel et al., 2015; Cormier and Woodgett, 2017; Patel and Woodgett, 2017); in addition, these kinases can become inhibited by way of several distinct mechanisms such as phosphorylation. These features allow the GSK3 paralogs to recruit unique signaling pathways and serve both redundant and non-redundant functions. It is therefore paramount to continue to define the specific physiological functions of GSK3α and GSK3β.

GSK3 is known to play a key role in synaptic plasticity (Hooper et al., 2007; Peineau et al., 2007; see reviews by Bradley et al., 2012; Jaworski et al., 2019; Sayas and Ávila, 2021), the mechanism by which neuronal connections are either strengthened or weakened and underlies learning and memory (Bliss and Collingridge, 1993; Collingridge et al., 2010). In particular, the processes of long-term potentiation (LTP) and long-term depression (LTD) of synaptic transmission have been studied overwhelmingly in the hippocampus owing to its crucial role in learning. A variety of approaches have elucidated hippocampal GSK3 function through the use of available pharmacology, genetic overexpression, knock-out (KO), knock-down (KD), or knock-in (KI). Each study has captured a different degree of targeting in terms of developmental time window, cell type, and model system. And while GSK3α and GSK3β have been studied in LTP and especially LTD, the physiological synaptic function of each paralog remains unclear.

Here we sought to determine the role of GSK3 paralogs in the process of LTP within CA1 neurons of adult hippocampal slices. We compared the effects of their conditional KO (cKO), using floxed (loxP flanked) *Gsk3a or Gsk3b* mouse lines and an established transgenic Cre line driven by *Camk2a* that mediates excision in CA1 beginning after weaning age. We found that cKOs had a ∼40–50% reduction in protein level for each targeted gene as judged by immunoblotting of hippocampus, without significant change in each corresponding paralog. Furthermore, immunohistofluorescence (IHF) confirmed that in each of the GSK3α and GSK3β cKOs, there was a selective signal reduction for the corresponding targeted protein in adult CA1 pyramidal neurons. GSK3α cKOs showed enhanced LTP at hippocampal CA3−CA1 synapses of slices obtained from 16-week-old mice, whereas deletion of GSK3β was without effect. Neither of the paralog-specific cKOs showed changes in basal synaptic function or the paired-pulse ratio (PPR), a measure of presynaptic probability of neurotransmitter release (Pr). These results further demonstrate how the two homologous kinases can function independently of one another. They show that LTP at adult CA3−CA1 synapses is specifically modulated by postsynaptic GSK3α, where its activity limits the magnitude of synaptic potentiation.

## Materials and Methods

### Mouse Lines

We generated experimental subjects from crosses of heterozygous floxed (f) B6.129-*Gsk3a^tm 1*Jrw*^* (Doble et al., 2007; MacAulay et al., 2007) or B6.129(CG)-*Gsk3b^tm 2*Jrw*^* mice (Patel et al., 2008). Each female breeder also carried a cre transgene introduced through crosses with Tg(Camk2a-cre)T29-1Stl/J mice (Jackson Laboratory, JAX stock #005359; see JAX website for extensive list of cited references) which mediate Cre/loxP recombination in the forebrain, postnatally beginning after weaning age in hippocampal CA1 pyramidal neurons (Tsien et al., 1996). All mouse lines were on a C57BL/6J genetic background. Tissue sections from subjects with *Gsk3a^f/f^*; Camk2a-cre or *GSK3b^f/f^*; Camk2a-cre genotypes are referred to as GSK3α or GSK3β cKOs (adult, CA1-selective), respectively. Comparisons were made to control Cre+ and floxed Cre- mice. Genotyping was performed as outlined in Supplementary Figure 1 which includes sample PCR results.

### Hippocampal Slice Preparation

Animals from mice aged 15.5–16.5 weeks were anesthetized using an isoflurane machine with vaporizer (Benson Medical Industries, Markham, ON, Canada) and decapitated using single edge blade in accordance with an Animal Use Protocol approved by The Centre for Phenogenomics (TCP; Toronto, ON, Canada) Animal Care Committee and conforming to the Canadian Council on Animal Care (CCAC) guidelines. Each brain was removed, the hemispheres were mounted and then sagittal slices prepared using a vibratome (VT1200S; Leica Microsystems, Richmond Hill, ON, Canada). Three slices from the dorsal side of the hippocampus (400 μm) were prepared for electrophysiology, and the remaining hippocampus was isolated and saved for biochemistry. Tissue slices were transferred to an incubation chamber (BSK12; Scientific Systems Design, Mississauga, ON, Canada) with artificial cerebral spinal fluid (ACSF) saturated with 95% O_2_ and 5% CO_2_. ACSF contained the following (mM): 26 NaHCO_3_, 10 D-Glucose, 124 NaCl, 1.25 NaH_2_PO_4_.H_2_O, 3 KCl, 2 CaCl_2_, and 2 MgCl_2_. Slices were allowed to recover at room temperature (21°C) for a minimum of 2 h before recordings were made.

### Electrophysiology

Extracellular recordings using dorsal hippocampal slices were performed in a SliceMate submerged chamber system (Scientifica, Uckfield, United Kingdom) maintained at 30°C and continuously perfused at 2.5 mL/min with ACSF. Standard glass microelectrodes (2–3 MΩ) were inserted into the CA1 stratum radiatum region of hippocampal slices to measure field excitatory postsynaptic potentials (fEPSPs) evoked with 0.1 ms biphasic current pulses (STG 4002; MCS, Kusterdingen, Germany) and a Pt:Ir stimulation electrode (FHC, Bowdoin, ME, United States). Recordings were amplified (Axopatch 1D, Molecular Devices, San Jose, CA, United States), digitized at 40 kHz (A/D) and recorded using Clampex (Molecular Devices, San Jose, CA, United States). N value indicates the number of animals, and n value indicates number of slices. For LTP experiments, the PPR (40 ms inter-pulse interval) was collected throughout (before and after the induction). After a stable baseline (0.033 Hz) of at least 20 min, LTP was induced using theta-burst stimulation (TBS) delivered at baseline stimulus current intensity. We either delivered a single episode of weak TBS (wTBS) or three episodes of spaced TBS (sTBS) at an inter-episode interval of 10 min (see Park et al., 2021). One episode of TBS comprised five bursts at 5 Hz, with each burst composed of five pulses at 100 Hz (25 pulses total).

### Biochemistry

The remainder of the whole-hippocampus tissue that was collected after the preparation of three dorsal slices for electrophysiology, was used for western blotting. The samples were lysed in cold RIPA buffer that contained phosphatase and protease inhibitor cocktail (New England BioLabs Canada Ltd., a Cell Signaling Technology [CST] product, Cat #5872). After centrifugation at 10,000 RCF for 30 min, the supernatant was collected, and protein concentration of the lysates determined with a bicinchoninic acid (BCA) kit (Pierce Biotechnology, Waltham, MA, United States). Proteins (20 μg per lane) were separated by SDS-PAGE and transferred onto PVDF membranes by Turbo Transfer (Bio-Rad Laboratories, Mississauga, ON, Canada).

A primary rabbit monoclonal antibody that recognizes both GSK3α and GSK3β (CST, Cat #5676) was used (1:1000, in TBST plus 5% non-fat dry milk). A mouse antibody against β-actin (1:10,000, CST, Cat #3700) was used for loading endogenous control; we verified that β-actin levels were not affected by conditional deletion of each of the two *Gsk3* paralogs (data not shown). The corresponding secondary antibodies were conjugated with HRP (1:10,0000, CST; anti-mouse IgG Cat #7074, or anti-rabbit IgG Cat #7076) or fluorescent StarBright Blue 700 (1: 20,000, Bio-Rad, Cat #10000068185D or 10000068187D). The experimenter was blinded to the genotype of each sample, which was run minimally in duplicate to produce one final averaged value associated with each sample. The signals were imaged with a ChemiDoc MP Imaging System (Bio-Rad) and analyzed with associated Image Lab software.

### Tissue Preparation for Staining

Mice aged 28 weeks were euthanized under isoflurane anesthesia. Brains were fixed by transcardial perfusion with 4% PFA. Briefly, the right atrium was accessed with procedure scissors and blunt needle was inserted through the left ventricle; the blood was rinsed out with 10 mL PBS and then 4% PFA was slowly perfused (20 mL) under low pressure through the systemic vasculature. Each brain was dissected out and postfixed in 4% PFA for 2 h, rinsed with PBS three times, transferred to 15% sucrose, and stored at 4°C overnight until each brain had sunk to the bottom of the holding tube. Both hemispheres were placed into a mold with Tissue Freezing Medium (Leica Biosystems Inc., Concord, ON, Canada, Cat #14020108926) and floated on the surface followed by rapid freezing with liquid nitrogen. Sagittal sections were cut at 20 μm thickness with a Leica cryostat and mounted to positive charged slides that were stored at −80°C until use.

### Immunohistofluorescence Detection and Imaging

Tissue sections on slides were washed with PBS for 3 × 5 min, antigen retrieved using boiling citrate buffer, pH 6.0, antigen retriever (Sigma-Aldrich Canada Co., Oakville, ON, Canada, Cat #C9999-1000ML) and microwave heated for an additional 5 min. The slides were cooled down to room temperature and washed with PBS 3 × 5 min. Sections were incubated with 5% normal donkey serum in 0.3% Triton X-100 in PBS for 1 h at room temperature to reduce non-specific staining. Next, they were incubated with primary antibodies, rabbit anti-GSK3α (1:50 CST, Cat #4818S), mouse anti-GSK3β (1:50, CST, Cat #9832S) diluted in 2.5% normal donkey serum containing 0.15% Triton X-100 in PBS at 4°C overnight. Slides were washed with PBS 3 × 5 min, incubated with secondary antibodies, donkey anti-rabbit conjugated with Alexa Fluor 594 (Jackson ImmunoResearch Inc, West Grove, PA, United States, Cat# 711-585-152) and donkey anti-mouse conjugated with Cy5 (Jackson ImmunoResearch Inc, Cat# 715-175-150) at 1:1000 diluted with PBS for 1 h at room temperature. The slides were washed with PBS for 3 × 5 min and mounted with a coverslip after adding 50 μL ProLong Glass Antifade Mountant (Thermo Fisher Scientific, Mississauga, ON, Canada, Cat# P36984). Images were captured with a 10 × and 60 × objective lens and appropriate filters using an A1R HD25 confocal microscope on a Ti2-E stand and running Elements software (Nikon Canada) at the OPTIMA facility (LTRI, Sinai Health).

### Analysis and Statistics

All electrophysiology and immunoblot experiments were carried out with the experimenter blinded to the genotype. Electrophysiology data were analyzed to obtain the rising slope of fEPSPs and fiber volley amplitude using Clampfit (Molecular Devices). For LTP experiments, including the PPR determination, responses were normalized to the last 10 min of baseline prior to TBS, and data are presented as the mean ± SEM. The number of slices (n) and subjects (N) analyzed are shown. Statistical significance was assessed using two-way ANOVA and unpaired *t*-tests (Prism; GraphPad Software, San Diego, CA, United States). The level of significance is denoted as follows: *p < 0.05, ^**^p < 0.01, and ^***^p < 0.001.

## Results

### Spatial Detection and Validation of the GSK3α and GSK3β cKO by Immunohistofluorescence

**FIGURE 1:**
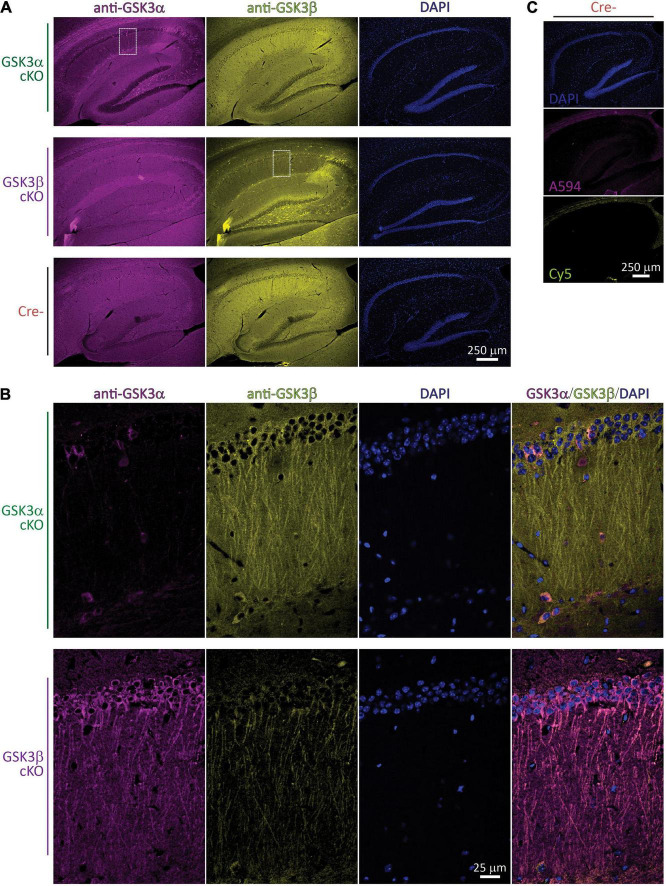
Spatial validation of GSK3α or GSK3β cKO by IHF. **(A)** Fluorescence images of brain sections through the hippocampus collected with a 10 × objective lens following staining with anti-GSK3α (revealed with Alexa 594) and anti-GSK3β (revealed with Cy5), as well as DNA using DAPI; the three columns on each row is from a single section imaged sequentially in the corresponding fluorescence wavelength channels. IHF from each of a GSK3α cKO, GSK3β cKO, and a Cre- subject are shown in rows. Note the prominent reduction in corresponding GSK3 staining within the CA1 region of each paralog cKO. **(B)** The white outlined zones in A were subsequently imaged with a 60 × lens and confocal optical resolution. The same fluorescence channels are presented, plus a final merged overlay in the final column. **(C)** Primary antibody was omitted, and images from the same Cre- subject were collected at the exact same settings as those used in **(A)**. **(A–C)** Images shown were selected from a sampling of *n* = 2–3 subjects per genotype, and are representative from a total of 36 images collected with a 10 × objective lens, and 82 images collected with a 60 × objective lens.

We evaluated GSK3 levels by IHF in each of the two conditional knockouts and controls. Sagittal brain sections through the dorsal hippocampus were prepared, antibodies against GSK3α and GSK3β were applied for multi-labeling, and images were acquired initially with a 10 × objective lens. Compared to the robust GSK3α and GSK3β signal in the CA1 region of controls, in each of the GSK3α and GSK3β cKOs there was a selective signal reduction for the corresponding targeted protein (Figure 1A). For instance, within the GSK3α cKO sample shown (Figure 1A, first row), the CA1 cell body layer showed dramatically reduced GSK3α staining. Similarly, in the GSK3β cKO sample shown in the second row, GSK3β was greatly reduced within the CA1 region. In each cKO, there were a few unaffected, brightly-labeled cells which were readily discernible against the reduced CA1 region signal intensity; these spared cells had their cell bodies situated in the stratum oriens, radiatum, as well as pyramidale layers, and based on their morphology appear to be interneurons.

The sub-regions outlined within Figure 1A (white dashed rectangle) were also imaged with a 60 × objective lens to achieve confocal resolution (Figure 1B). In these optical sections, the specific loss of targeted GSK3 signal within most of the CA1 excitatory neurons is evident. While there is less out-of-focus fluorescence captured, the location of individual somata could still be identified by the DAPI staining of nuclear DNA. For instance, in the GSK3α cKO area sampled at the higher magnification (Figure 1B, upper row) there is a field of > 50 stratum pyramidale cells with ∼5 spared cells labeled with anti-GSK3α, all of which also expressed GSK3β (see relevant channels in columns, as well as merged image in the last column); most of these spared cells did not appear to show any long apical dendrites stained and therefore may not be excitatory neurons. Analogously, in the GSK3β cKO subregion sampled at the higher resolution (Figure 1B, lower row image set), the somatic GSK3β levels are nearly eliminated, though some residual dendritic signal persisted; amongst the various CA1 cell layers, there are likewise a low number of unaffected GSK3α-positive cells expressing both paralog products.

Secondary-only controls were carried out (primary antibodies omitted) and are presented in Figure 1C. These controls produced nearly no signal within the CA1, suggesting the anti-GSK3 antibodies are effectively the main source of the signals observed in Figures 1A,B. Both Cy5 (anti-mouse targeting anti-GSK3β) and Alexa594 (anti-rabbit targeting anti-GSK3α revealed extra-hippocampal bands of staining corresponding to myelinated fibers of the corpus callosum. For Alexa594, there was some very weak additional staining in stratum lacunosum-moleculare and the hilus.

### GSK3α and GSK3β cKOs Show Reductions in Corresponding Protein Expression

**FIGURE 2:**
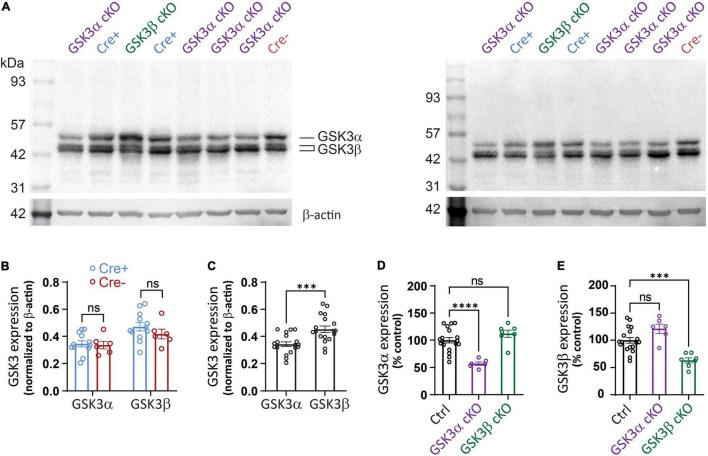
Western blot detection of targeted protein reduction in GSK3α or GSK3β cKO. **(A)** Western blot sample results using hippocampal lysates from tissue studied in Figures 3–5. Lysates from GSK3α cKO, GSK3β cKO, Cre+, and Cre- were loaded by the experimenter blinded to genotype and then the data was averaged from 2 to 4 technical replicates per slice. **(A)** Shows two of the technical replicates for samples obtained from the same 8 mice. Blots were probed initially for GSK3 and subsequently for β-actin. **(B)** Similar levels of expression of GSK3α and GSK3β when Cre+ and Cre- mice are compared. Note that both isoforms of GSK3β were detected and included in the analysis. **(C)** Quantification shows a higher signal for GSK3β compared to GSK3α for pooled control samples. **(D)** Reduced GSK3α expression in the GSK3α cKOs compared to control (Ctrl) samples, with no significant change in level of the GSK3β paralog. **(E)** Reduced GSK3β expression in the GSK3β cKOs compared to control (Ctrl) samples, which was associated with no significant change in level of the GSK3α paralog. Data for **(B)** were analyzed with two-way ANOVA; effect of Cre+ vs. Cre- [*F*(1,32) = 1.119, *p* = 0.2981; effect of GSK3α vs. GSK3β: *F*(1,32) = 12.35, *p* = 0.013; effect of interaction: *F*(1,32) = 0.563, *p* = 0.4584, *N* = 18.] Data shown in **(C–E)** were analyzed with an unpaired Student *t*-test. ***p < 0.001, ****p < 0.0001, ns, not significantly different. *N* = 18 for control, *N* = 6 for GSK3α cKO, *N* = 7 for GSK3β cKO.

We also employed western blotting to quantify the effectiveness of the two cKOs, including any potential compensation. Immunoblotting experiments were carried out using tissue lysates prepared from the extra, whole hippocampal slices that were collected for the electrophysiology work. Two exemplar blots are shown in Figure 2A, which are technical replicates from a random array of subjects (16 blots in total were analyzed with 8-9 sample lanes each). For each unique sample, one value (N) was determined based on an average from all technical replicates (*n* = 2-4). Each paralog product was distinguished by the unique migration band sizes.

Our first comparison determined whether introduction of the cre transgene influenced GSK3 expression; both GSK3α and GSK3β expression were unchanged across the Cre+ and Cre-lacking (Cre-) control samples (Figure 2B), confirming that the cre transgene did not alter protein levels of the GSK3 paralogs. As we employed a single antibody that detects both isoenzymes, it became evident that there was a trend toward higher abundance of GSK3β. We next pooled data from all control groups (floxed, Cre-, and wildtype) and indeed found a small, but statistically significant higher level of signal for GSK3β over GSK3α (Figure 2C). We cannot exclude the possibility that the anti-GSK3 antibody does not bind equally well to both paralogs, though we can conclude that both paralogs are expressed abundantly in hippocampal tissue.

The western blot analysis showed a nearly 50% reduction in GSK3α protein and over 40% reduction in GSK3β protein in each of the respective cKO (Figures 2D,E). In each cKO, compared to control samples, there was a statistically non-significant 10–20% increase in protein level of the non-targeted paralog product. The results suggest that in each of the GSK3 cKOs, there are no appreciable compensatory changes in non-homologous protein expression. As GSK3 is expressed in other cell types and hippocampal sub-regions not expressing the Cre recombinase, it was not surprising that expression was not eliminated. Regardless, the substantial reduction in protein supports the argument that our genetic knockout strategy was effective and that both paralogs exist within adult hippocampus, which is consistent with the IHF results that used paralog-selective antibodies.

### CA3−CA1 Neurotransmission Is Unaffected by GSK3α or GSK3β Deletion

**FIGURE 3:**
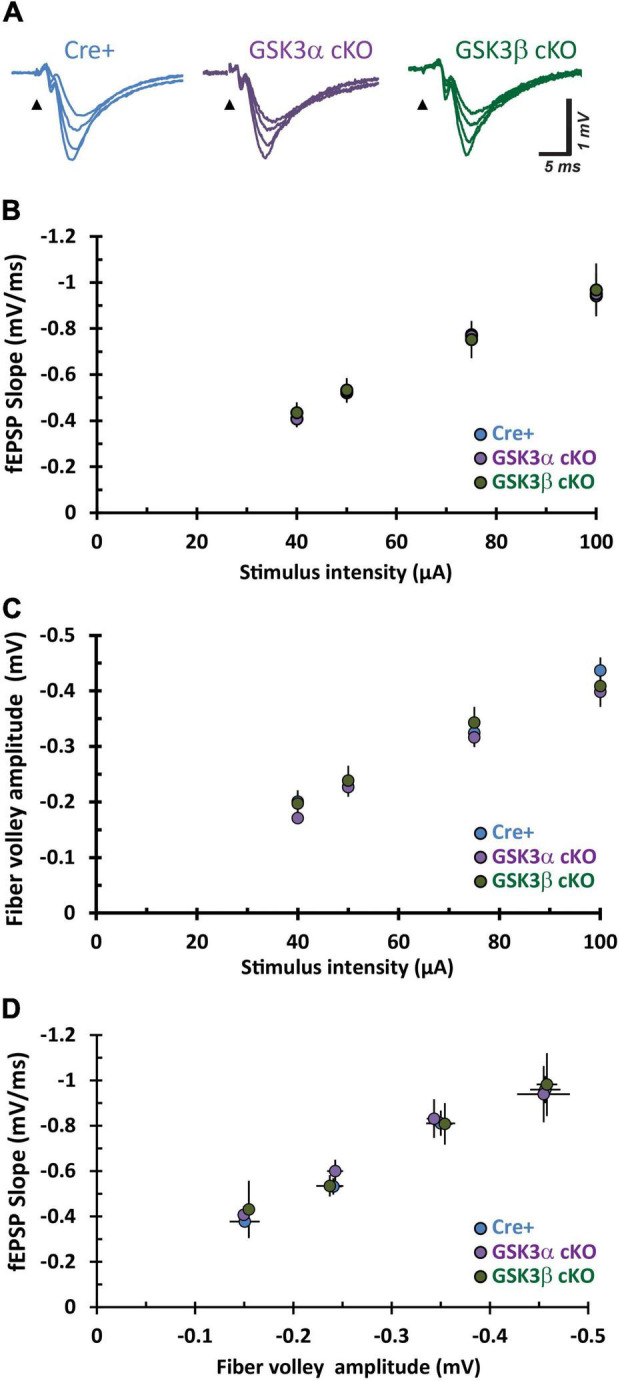
Adult CA3–CA1 basal synaptic function is unaffected by cKO of GSK3α or GSK3β. **(A)** Sample fEPSP traces from hippocampal CA1 slices evoked by test pulse stimulation of Schaffer collaterals at increasing current stimulation (40, 50, 75, and 100 μA all superimposed). **(B–D)** Input – output function of synaptic transmission evaluated in three different pairwise comparisons for stimulus current intensity, fEPSP slope, and fiber volley amplitude. The repetitions for Cre-positive (Cre+), GSK3α and GSK3β cKO are *n* = 7, *N* = 7; *n* = 12, *N* = 6; and *n* = 7, *N* = 6, respectively.

We then explored whether deletion of either GSK3 paralog in CA1 neurons of murine hippocampus impacted synaptic transmission. Synaptic responses in the stratum radiatum of adult tissue slices were evoked by applying test stimuli to the Schaffer collateral-commissural pathway in CA3 (Figure 3A). Increasing the current pulse amplitude yielded a corresponding rise in the fiber volley amplitude and slope of the evoked fEPSP slope in all three groups. Pairwise comparisons for these three measures revealed relationships that did not differ between the Cre-positive (Cre+) control, GSK3α cKO, and GSK3β cKO groups (Figures 3B–D). The data indicated that genetic elimination of either GSK3α or GSK3β expression in CA1 pyramidal neurons, after development, does not affect the number of functional axons or their ability to evoke neurotransmitter release and postsynaptic responses at adult CA3−CA1 synapses.

### Conditional Deletion of GSK3α, but Not 3β, Enhances Long-Term Potentiation

**FIGURE 4:**
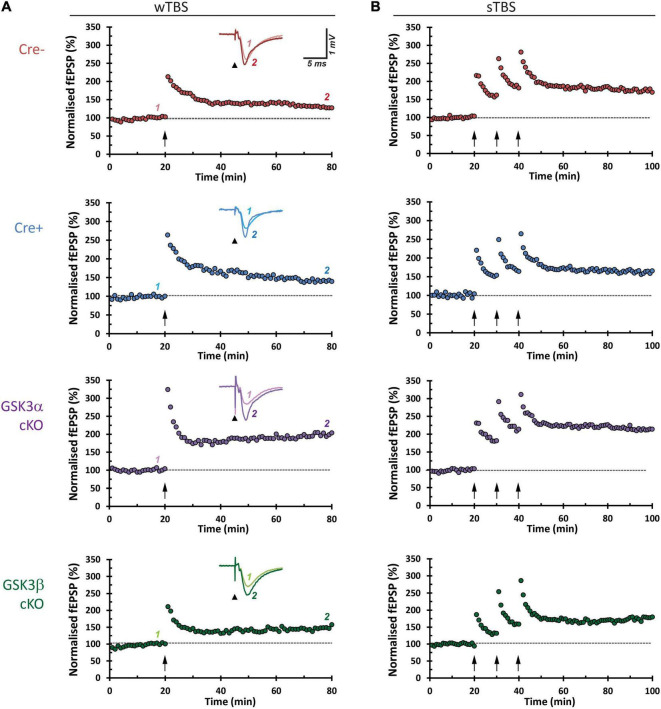
Representative hippocampal slice LTP time-course plots. Each row shows an experimental LTP result from a Cre-, Cre+, GSK3α cKO, and GSK3β cKO hippocampal slice. **(A)** The left column panels are from LTP experiments on samples that received a 25-pulse, weak, theta-burst stimulation (wTBS) induction protocol (arrow, time = 20 min). Insets show averaged synaptic response (5 traces) obtained by the first of paired pulses of current stimulation (arrowhead, stimulus pulse and corresponding artifact), before (1) and after LTP (2), superimposed. **(B)** Sample results from experiments that received the sTBS (3 × TBS) induction protocol.

**FIGURE 5:**
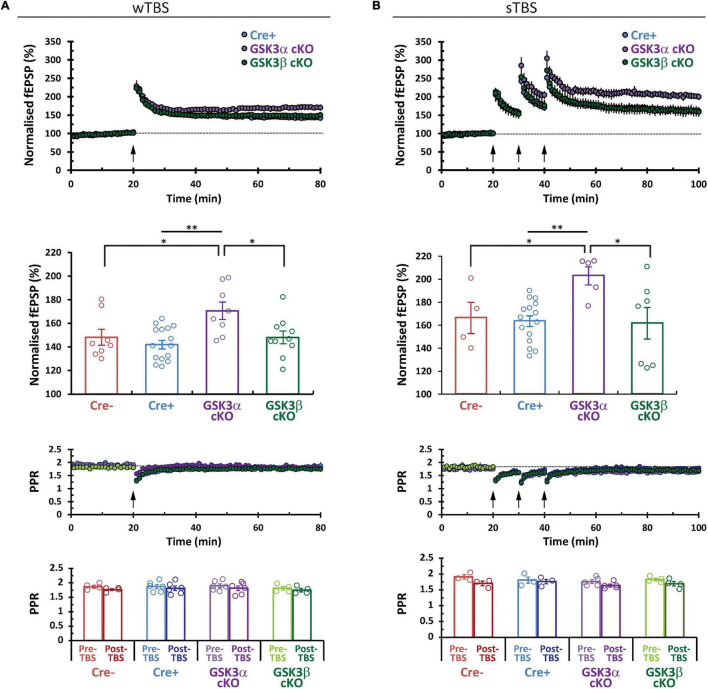
GSK3α cKOs show a selective increase in CA3–CA1 LTP. Comparison of the averaged LTP data sets for **(A)** wTBS and **(B)** sTBS (3 × TBS) induction protocols. Corresponding bar plot summary (second row) shows the LTP level achieved at the last 10 min of each experimental series (normalized to the 10 min of baseline prior to LTP induction). Statistics for wTBS: Cre+ (*N* = 9, *n* = 15) vs. α cKO (*N* = 6, *n* = 8), **P < 0.01. Cre- (*N* = 5, *n* = 8) vs. GSK3α cKO, *P < 0.05; α cKO vs. β KO (*N* = 7, *n* = 10), *P < 0.05. Statistics for sTBS: Cre+ (*N* = 10, *n* = 15) vs. α cKO (*N* = 4, *n* = 5), **P < 0.01; Cre- (*N* = 3, *n* = 4) vs. α cKO, *P < 0.05; α cKO vs. β KO (*N* = 5, *n* = 7), *P < 0.05. The time course of the PPR is shown and averages are summarized in the bar plots (lower two rows of data).

CA3−CA1 synapses display several forms of NMDA receptor-dependent LTP. Two induction paradigms that produce mechanistically distinct types of LTP were compared: (1) a weak theta-burst stimulation (wTBS) protocol, and (2) three rounds of spaced TBS (sTBS), which produces an additional form of LTP that is dependent on the recruitment of PKA, calcium-permeable AMPA receptors, and protein synthesis (Park et al., 2021). In both paradigms there is an initial short-term potentiation (STP) that constitutes a mechanistically distinct form of synaptic plasticity (France et al., 2022). Example time course plots for wTBS- and sTBS-LTP experiments, from each of the four groups, are shown in Figure 4. The averaged datasets revealed that compared to the two control groups, GSK3α cKO slices have significantly enhanced LTP in both the wTBS (Figure 5A) and sTBS (Figure 5B) experiments. In contrast, the GSK3β KO slices were not different from controls in terms of synaptic plasticity induced by either the wTBS (Figure 5A) or sTBS (Figure 5B) paradigms. This effect on LTP in the GSK3α KO was not associated with any alteration in the level of STP. We also plotted the averaged PPR (40 ms inter-pulse interval) collected during the time-course of LTP experiments, which revealed no differences by genotype nor TBS treatment, neither at baseline nor at the end of the LTP experiment (Figures 5A,B, lower two rows of data). The transient decrease in PPR, that reflects an increase in the probability of neurotransmitter release during STP, was also similar between genotypes. These results show that in the adult CA1, GSK3α in excitatory neurons limits the extent of LTP, but not STP, achieved; accordingly, the underlying mechanism is unlikely to involve changes in the probability of transmitter release.

## Discussion

### Conditional GSK3α or GSK3β Deletion in CA1

To examine the function of GSK3 in the hippocampus, we crossed each of our *Gsk3a* and *Gsk3b* floxed mice to an established transgenic cre line (T29-1) that excises DNA in CA1 pyramidal neurons after weaning age. This strategy avoided the impact of suppressing GSK3 activity during neurodevelopment, allowing normal brain organization. We carried out IHF, which revealed that CA1 dendrites express both paralogs, and that each of the GSK3α and GSK3β cKOs yielded a selective signal reduction for the corresponding targeted protein. In the CA1 region of each cKO, there were cells that were spared from GSK3 deletion, which appeared to be interneurons based on morphology and the location of their cell bodies.

In the case of the GSK3β cKO, IHF showed that most of the signal at the CA1 somata was eliminated. The signal in the CA1 dendrites was also greatly reduced, though there was a residual signal. While we cannot rule out low levels of remaining protein, it is also possible that the antibody weakly detected a non-specific target expressed in the dendrites alone. We also carried out western blotting quantification of tissue from hippocampus, which confirmed the specific reduction of the correct protein for each of the targeted genes, without any significant changes in protein expression of each corresponding paralog. Thus, we were able to selectively evaluate the consequences of conditionally deleting each of the GSK3 paralogs in CA1 excitatory neurons at an early adult stage.

In hippocampal slices from 16-week-old adult cKO mice, we found that LTP was elevated in the GSK3α cKO, but LTP in hippocampal slices from GSK3β cKO animals remained unaffected. As we did not detect any changes in PPR, functional axons, or transmitter release, the effect is unlikely to involve changes in the probability of transmitter release nor in other presynaptic factors. Instead, the underlying mechanism by which GSK3α modulates synaptic plasticity implicates post-synaptic mechanisms.

There are an increasing number of reports that have implicated hippocampal GSK3 in a variety of behaviors that evaluated cognition, anxiety, stress and depression (e.g., Latapy et al., 2012; Hui et al., 2018; Lee et al., 2022). Constitutive GSK3α KOs have been studied and found to have a range of morphological and neurobehavioral phenotypes including impaired fear memory (Kaidanovich-Beilin et al., 2009). In contrast to GSK3β nulls, heterozygous mice survive but display anxiety and deficient memory reconsolidation (Bersudsky et al., 2008; Kimura et al., 2008). Furthermore, we know that the removal of additional GSK3 alleles has a compounding effect on the brain; we originally generated constitutive GSK3α KOs and also floxed *Gsk3b* mice that we crossed to a nestin-cre deleter line and found major brain developmental malformations (Kim et al., 2009). The consequences of deleting each and also both paralogs was subsequently studied in the context of identification of unique substrate phosphorylation in the cortex (Soutar et al., 2010).

### Hippocampal Synaptic Studies That Block *Gsk3* Expression in Mice

Several studies have used genetically modified mouse models, in particular to study GSK3β function (see reviews by Kaidanovich-Beilin and Woodgett, 2011; Jaworski et al., 2019). For instance, the same line of floxed *Gsk3b* mice that we have used were crossed with the M34 *Camk2*-cre deleter line to compare behavioral phenotypes with those found in our global GSK3β KOs (Latapy et al., 2012). Interestingly, a subsequent study found that expression of the neuronal calcium sensor-1 (NCS-1) was elevated in the frontal cortex of the GSK3β cKOs (Magno et al., 2020). Regulation of NCS-1 is relevant to synaptic plasticity since we have previously shown that NCS-1 overexpression enhances LTP in the dentate gyrus (Saab et al., 2009), whereas NCS-1 KOs have reduced LTP in CA1 (Ng et al., 2020) and LTD in perirhinal cortex is blocked after RNAi or dominant-negative mutant NCS-1 (Jo et al., 2008). KO of GSK3α may lead to elevated NCS-1 and enhanced LTP in the CA1. Future experiments will be needed to test this hypothesis.

Adult mice with sparse forebrain cKO of GSK3β have yielded roles in dendritic spine structure and function (Ochs et al., 2015). SLICK-V mice that express a tamoxifen-inducible Cre recombinase and YFP, driven by two copies of the thymocyte differentiation antigen 1 (Thy1) promotor, were crossed with our floxed *Gsk3b* mice to yield a very low proportion of KO neurons in CA1. The authors found reduced dendritic spine density in CA1 apical dendrites of GSK3β KO neurons.

### Neuronal *Gsk3a* Gene Disruption

Expression of GSK3α has been conditionally deleted in neurons, by crossing our floxed *Gsk3a* mice to a Cre recombinase line driven by the *Thy1* promoter (Maurin et al., 2013). Western blotting of hippocampus tissue revealed an ∼80% reduction in total *Gsk3a* protein, with a modest but significant increase in GSK3β. CA3−CA1 synaptic responses and synaptic fatigue were slightly increased and LTP reduced in the GSK3α cKOs. These differences to our present findings may relate to the properties of the cre deletor. The JAX strain #006143 Tg(Thy1-cre)1Vln cre line expresses active cre from an early postnatal stage, including embryonic stages in some brain regions. The *Thy1.2* promotor is known to generate a wide array of transgene expression patterns beginning in very early development, and not only in pyramidal cells but also inhibitory neurons and even some astrocytic and non-pyramidal cells (Feng et al., 2000; Porrero et al., 2010). The wider temporal and spatial expression pattern is consistent with the more extensive reduction in protein level reported in the cKO deleted via the *Thy1*-cre transgenic approach.

### Knockdown of *Gsk3a* and *Gsk3b*

Recently, an shRNA approach was employed to KD each of *Gsk3a* and *Gsk3b* (Draffin et al., 2021). In patch-clamped CA1 neurons of rat organotypic slice cultures, KD of *Gsk3a*, but not *Gsk3b*, resulted in complete block of NMDAR-mediated LTD. KD of *Gsk3b* or overexpression of EGFP-tagged versions of either paralog led to a 20% depression of AMPAR-mediated currents. There were no effects of overexpression or KD of either paralog on NMDAR-mediated synaptic transmission. It is possible that changes in LTD efficacy can shift the balance of synaptic plasticity toward LTP. Additionally, these effects may be specific to the developmental stage and slice culture model; future work with a cre deletor line that can excise at an early postnatal stage may be useful to evaluate synaptic function including LTD using *ex vivo* slices.

### Genetic GSK3 Overexpressing Rodent Models

When human GSK3β is conditionally overexpressed in the forebrain of mice throughout life, LTP is inhibited at CA3−CA1 synapses (Hooper et al., 2007). Interestingly, when the mice were pre-treated with a lithium diet for 3−4 weeks, LTP was normal. These Tet/GSK3β transgenic mice were made by crossing a *CMV*-Tet line encoding myc-GSK3β and β-galactosidase, with mice that express the tetracycline-regulated transactivator (tTA) under the control of a CamKIIα promotor (Lucas et al., 2001). Tet/GSK3β mice reveal neuropathological aspects of AD and have also been shown to have spatial learning deficits (Hernández et al., 2002).

Transgenic mice which overexpress constitutively active GSK3β (S9A, driven by Thy-1 promotor) have also been generated and studied in several articles (see review by Jaworski et al., 2019). Phosphorylation defective GSK3β-S9A expressing mice show reduced late LTP, enhanced LTD, and impaired long-term memory (Dewachter et al., 2002); the authors suggested that the unique effect on late LTP that contrast to the immediate effects on LTP observed in the Tet/GSK3β slices mentioned further above (Hooper et al., 2007), may be due to the relatively lower level of overexpression. It is difficult to compare the results from an overexpression approach that clearly demonstrates the potential for GSK3β to affect LTP, with that of our current neuronal KO study. We induced and recorded LTP for an hour in our GSK3β cKO and found no effect within this time frame, using two paradigms that induce mechanistically unique forms of LTP.

### Constitutively Active GSK3 Knockin Mice (Lacking Inhibitory Regulation)

A genetic approach was used to generate KI mice encoding constitutively active GSK3α (Ser21) or GSK3β (Ser9) under conditions of normal expression levels, leading to the finding that GSK3β is the major regulator of glycogen synthase in skeletal muscle (McManus et al., 2005). Furthermore, CA1 slices from the same mouse model were subsequently studied and the GSK3α KI showed reduced paired-pulse ratios, suggesting an increase in the probability of transmitter release (Shahab et al., 2014). In addition, there was a near elimination of both LTP and LTD in the GSK3α KIs. The GSK3β KI did not reveal any synaptic changes over wild-type controls. Therefore, preventing phosphorylation of Ser21 on GSK3α (which serves to inhibit the kinase), blocked synaptic plasticity bi-directionally.

Double GSK3α/3β KIs (Ser21/9) mice have been studied and found not to have any changes in ventral hippocampus CA3−CA1 synaptic function, nor in LTP (Polter et al., 2010). However, LTD was abolished, an effect that was suggested to involve heightened stress. The same GSK3 KI model (but on a C57BL/6J genetic background) was employed in a separate study that focused on the discovery that wildtype mice show a circadian regulation of GSK3β phosphorylation in the CA1, that becomes elevated during the night (Besing et al., 2017). Moreover, there was enhanced CA3−CA1 LTP in wildtype slices obtained during the night versus the day. In double GSK3 KI mice, the same night/day difference in extent of LTP persisted, however the absolute level of LTP achieved was enhanced at both night and day. Interestingly, CHIR-99021, an inhibitor of both GSK3 paralogs, reduced LTP magnitude only during the night. Regardless, GSK3 phosphorylation is regulated throughout the day and modulates LTP in CA1. It is unknown, but plausible that rhythmic GSK3 activity also regulates LTD.

### Does GSK3β Play Any Critical Role in CA1 Long-Term Potentiation?

We did not detect any changes in CA3−CA1 basal synaptic function or LTP in slices from GSK-3β cKO subjects. GSK3β does exist in CA1 neurons and has been effectively reduced in our study and in other genetic approaches (Latapy et al., 2012; Maurin et al., 2013; Ochs et al., 2015; Figures 1, 2E herein). Moreover, it has been shown that GSK3β reduction affects CA1 dendritic spine morphology and excitatory postsynaptic activity (Ochs et al., 2015) which has also been documented in pyramidal neurons of the medial prefrontal cortex following somatic *Gsk3b* KO (Khlghatyan et al., 2018). The latter study also demonstrated an increase in the AMPA receptor rectification index, which suggests an increase in calcium-permeable AMPA receptors. We have previously demonstrated that transient insertion of calcium-permeable AMPA receptors underlies LTP2, a type of synaptic plasticity that can be induced by spaced TBS (Park et al., 2019, 2021).

In another study on cortical neurons lacking GSK3β targeted through crosses of our floxed mice with *Drd2*-cre, there were no changes in AMPAR-mediated spontaneous and miniature events (Li et al., 2020). Instead, NMDAR-mediated function was enhanced, a finding that was further substantiated using direct injections of viral *Syn*-cre into floxed *Gsk3b* mice. Moreover, an induction paradigm that normally doesn’t elicit LTP (due to intact GABAergic inhibition), was able to generate LTP in the *Drd2*-cre GSK3β cKOs; LTD was also evaluated, which in contrast to control mice, could not be induced in the cKOs.

It is known that following LTP-inducing stimuli in the CA1 and dentate gyrus, GSK3β becomes phosphorylated at Ser9 for at least an hour (Hooper et al., 2007; Peineau et al., 2007; Cai et al., 2008), which will inhibit the kinase. We have previously suggested that a transient inhibition of GSK3β activity may preserve learning during LTP by preventing synapses from undergoing LTD (Peineau et al., 2007). Several articles have implicated GSK3β in LTD (reviewed by Jaworski et al., 2019), a normal physiological process that allows for resetting of synaptic strength, effectively enables bi-directional plasticity, and mediates several forms of memory (Collingridge et al., 2010).

It is possible that the GSK3β paralog plays a more important in neuronal excitability and damage. GSK3β controls many mechanisms that mediate excitability, which if dysregulated can trigger seizures and neurodegeneration (see review by Jaworski, 2020). While interference with GSK3β reduces synaptic loss and tau-associated pathology, both GSK3 paralogs mediate LTD processes (reviewed by Sayas and Ávila, 2021); excessive LTD is relevant here because this mechanism may lead to synaptic loss, cell death, and a range of disorders (Collingridge et al., 2010). It is worth mentioning that FMRP knockouts show elevated GSK3 and mGluR5 activity (Min et al., 2009), and that BRD0705, an inhibitor with some selectivity toward GSK3α, corrects the hyperexcitability in visual cortex slices, as well as the elevated protein synthesis in hippocampal slices (McCamphill et al., 2020). Therefore, GSK3 regulates the two key neuronal processes of synaptic plasticity and neuronal excitability, in the context of both physiological regulation and aberrant function leading to brain disorders.

In summary, several different approaches (pharmacology, overexpression, KI, KD, and KO) have been used to distinguish the roles of the GSK3 paralogs in synaptic plasticity at CA1 synapses, but no clear picture has emerged. This motivated us to employ a conditional KO approach where we found a clear role for GSK3α, but not GSK3β, in limiting the magnitude of LTP in adult mice. Further work is needed to determine the selective mechanism by which GSK3α mediates this effect.

## Conclusion

The two GSK3 paralogs have a range of functions both inside and outside the brain throughout life. It is critical to understand the normal physiological functions of GSK3, especially since its modulation is being considered for treatments in a variety of disorders. Here we took a reductionist approach to study the effect of deleting each GSK3 paralog specifically in adult neurons and recorded CA3−CA1 synaptic responses; this prototypic hippocampal circuit displays LTP, a crucial form of synaptic plasticity that is required for learning and memory. Given that interference with only *Gsk3a* expression resulted in enhanced LTP, we speculate that GSK3α activity may serve to limit LTP in excitatory CA1 neurons of adult mice.

These findings are consistent with a specific role of GSK3α in LTD (Shahab et al., 2014; Draffin et al., 2021). One possibility is that in adult mice, LTD limits the magnitude of LTP and that the enhanced LTP observed in the KO is due to the acute elimination of this LTD. Consistent with this notion, we have recently observed NMDAR-LTD in adult anesthetized mice that is eliminated by a GSK3 inhibitor, CT99021 (Lee et al., 2022). Interestingly, in this study, CT99021 enhanced learning and memory, which is consistent with the enhanced LTP that we report here. Due to the potential importance of GSK3 paralogs as therapeutic targets (Georgievska et al., 2013), in the absence of highly paralog-selective inhibitors, it will be important to combine pharmacological and genetic approaches in future mechanistic studies.

## Data Availability Statement

The original contributions presented in the study are included in the article/Supplementary Material, further inquiries can be directed to the corresponding author/s.

## Ethics Statement

The animal study was reviewed and approved by TCP (The Centre for Phenogenomics) Animal Care Committee (ACC).

## Author Contributions

AEA carried out all of the electrophysiology based on pilot data provided by TM. TM generated, genotyped, and provided the mice with assistance from ER. GL performed the western blotting. FJ carried out the immunohistofluorescence and post-experiment genotype validation. JG, JW, and GC designed and directed the research. AEA and GL analyzed the data, prepared the figures, and wrote the methods. JG, CB, JW, and GC interpreted the data and wrote the manuscript. All authors reviewed and edited the manuscript.

## Conflict of Interest

The authors declare that the research was conducted in the absence of any commercial or financial relationships that could be construed as a potential conflict of interest.

## Publisher’s Note

All claims expressed in this article are solely those of the authors and do not necessarily represent those of their affiliated organizations, or those of the publisher, the editors and the reviewers. Any product that may be evaluated in this article, or claim that may be made by its manufacturer, is not guaranteed or endorsed by the publisher.
