# Supplementary material for: Specific Role for GSK3α in Limiting Long-Term Potentiation in CA1 Pyramidal Neurons of Adult Mouse Hippocampus
Source: Front Mol Neurosci. 2022 Jun 17;15:852171. doi: 10.3389/fnmol.2022.852171 (PMC9247355; doi:10.3389/fnmol.2022.852171)
Supplement: Supplementary file 1 [file Data_Sheet_1.PDF]

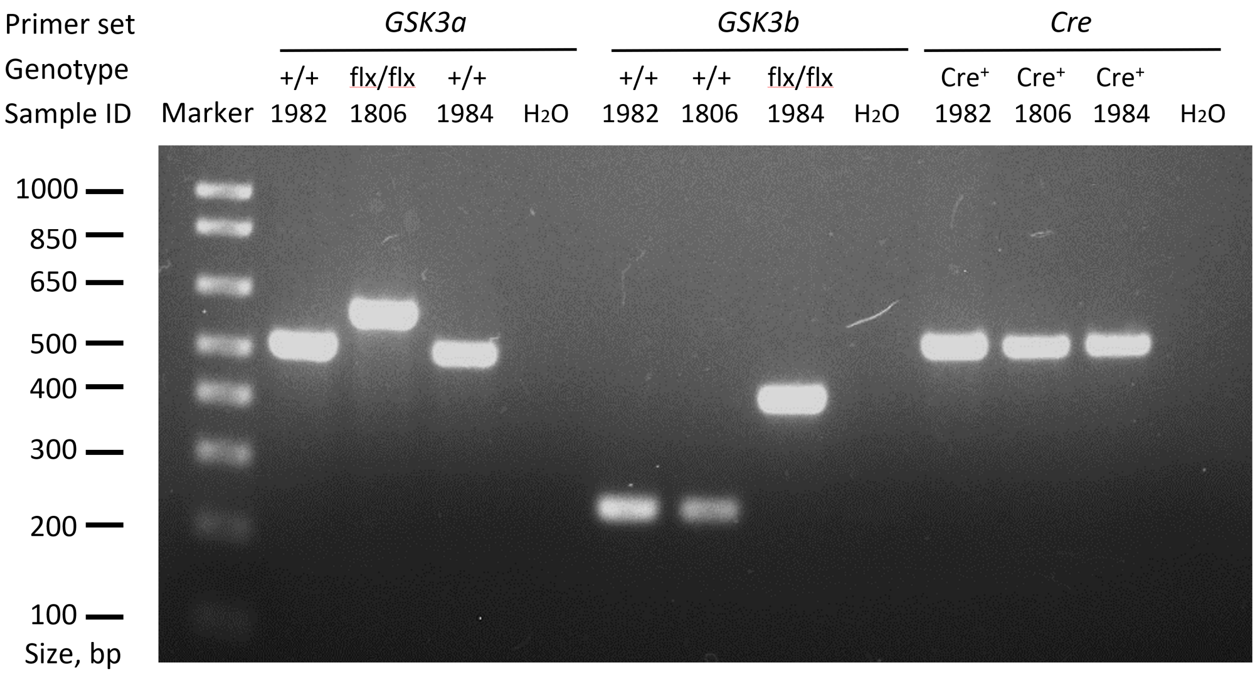

Supplementary Figure 1. PCR genotyping of the GSK3α and GSK3β cKO. Example agarose gel electrophoresis stained with SYBR Safe (Invitrogen) showing PCR genotyping results for genomic DNA obtained from a selection of samples and negative water controls. HotSHOT DNA preparation was carried out as described [1]. An alkaline lysis reagent with 25 mM NaOH, 0.2 mM disodium EDTA and a neutralizing reagent with 40 mM Tris-HCl were prepared without adjusting the pH. Mouse tail snips or ear biopsy samples were digested with 50 μL alkaline solution at 95 °C for 20 min and then 50 μL of neutralization buffer was added. Primers for the PCR and expected amplicons are listed below. A touch-down PCR program was employed. Step 1: 95 °C for 5 min. Step 2: 95 °C for 30 s. Step 3: 65 °C for 30 s and every cycle decreased 0.5 °C. Step 4: 68 °C for 1 min. Step 5: go to step 2 for 10 cycles. Step 6: 95 °C for 30 s. Step 7: 60 °C for 30 s. Step 8: 72 °C for 1 min. Step 9: go to step 6 for 28 cycles. Step 10: 72 °C for 15 min. Step 11: keep at 4 °C for 12 h. PCR master mixture included DNA fast extract 2× HS-Red Taq Mix (WISENT Inc., Canada; cat #801-200-HS). The PCR products were examined on a 2 % agarose gel. +/+ = WT, wildtype; floxed = flx.

Allele	Name	Sequence (5'-3')	Amplicon size
GSK3a	GSK3aFlx_F	CCCCCACCAAGTGATTTCACTGCTA	+ : 500 bp<br>flx : 600 bp
	GSK3aFlx_R	CTTGAACCTTTTGTCCTGAAGAACC	
GSK3b	GSK3bFlx_F	GCCATCAAGAAAGTTCTACAGGA	+ : ~250 bp<br>flx : ~450 bp
	GSK3bFlx_R	GCTGAAGTCCAGAGCAAGTCT	
Cre	Cre553-72_F	TCGCAAGAACCTGATGGACA	Cre+: 539 bp
	Cre1072-92_R	AGCGTTTTCGTTCTGCCAATA	

F: forward primer. R: reverse primer

1. Truett, G.E., et al., *Preparation of PCR-quality mouse genomic DNA with hot sodium hydroxide and tris (HotSHOT).* Biotechniques, 2000. **29**(1): p. 52, 54.
